# Colchicine in patients with acute ischaemic stroke or transient ischaemic attack (CHANCE-3): multicentre, double blind, randomised, placebo controlled trial

**DOI:** 10.1136/bmj-2023-079061

**Published:** 2024-06-26

**Authors:** Jiejie Li, Xia Meng, Fu-Dong Shi, Jing Jing, Hong-Qiu Gu, Aoming Jin, Yong Jiang, Hao Li, S Claiborne Johnston, Graeme J Hankey, J Donald Easton, Liguo Chang, Penglai Shi, Lihua Wang, Xianbo Zhuang, Haitao Li, Yingzhuo Zang, Jianling Zhang, Zengqiang Sun, Dongqi Liu, Ying Li, Hongqin Yang, Jinguo Zhao, Weiran Yu, Anxin Wang, Yuesong Pan, Jinxi Lin, Xuewei Xie, Wei-Na Jin, Shuya Li, Siying Niu, Yilong Wang, Xingquan Zhao, Zixiao Li, Liping Liu, Huaguang Zheng, Yongjun Wang

**Affiliations:** 1 Department of Neurology and China National Clinical Research Center for Neurological Diseases, Beijing Tiantan Hospital, Capital Medical University, Beijing, China; 2 Clinical Center for Precision Medicine in Stroke, Capital Medical University, Beijing, China; 3 Department of Neurology, University of California, San Francisco, CA, USA; 4 Medical School, University of Western Australia, Perth, WA, Australia; 5 Perron Institute for Neurological and Translational Science, Perth, WA, Australia; 6 Department of Neurology, Liaocheng Third People’s Hospital, Shandong, China; 7 Department of Neurology, Yantai Penglai Traditional Chinese Medicine Hospital, Shandong, China; 8 Department of Neurology, The Second Affiliated Hospital of Harbin Medical University, Heilongjiang, China; 9 Department of Neurology, Liaocheng People’s Hospital, Shandong, China; 10 Department of Neurology, The People’s Hospital of Qihe County, Shandong, China; 11 Department of Neurology, Qinghe People’s Hospital, Hebei, China; 12 Department of Neurology, The Fourth People’s Hospital of Hengshui, Hebei, China; 13 Department of Neurology, Zibo Municipal Hospital, Shandong, China; 14 Department of Neurology, Hejian People’s Hospital, Hebei, China; 15 Department of Neurology, Suixian Chinese Medicine Hospital, Henan, China; 16 Department of Neurology, Jiyuan Hospital of TCM, Henan, China; 17 Department of Neurology, Weihai Wendeng District People's Hospital, Shandong, China

## Abstract

**Objectives:**

To assess the efficacy and safety of colchicine versus placebo on reducing the risk of subsequent stroke after high risk non-cardioembolic ischaemic stroke or transient ischaemic attack within the first three months of symptom onset (CHANCE-3).

**Design:**

Multicentre, double blind, randomised, placebo controlled trial.

**Setting:**

244 hospitals in China between 11 August 2022 and 13 April 2023.

**Participants:**

8343 patients aged 40 years of age or older with a minor-to-moderate ischaemic stroke or transient ischaemic attack and a high sensitivity C-reactive protein ≥2 mg/L were enrolled.

**Interventions:**

Patients were randomly assigned 1:1 within 24 h of symptom onset to receive colchicine (0.5 mg twice daily on days 1-3, followed by 0.5 mg daily thereafter) or placebo for 90 days.

**Main outcome measures:**

The primary efficacy outcome was any new stroke within 90 days after randomisation. The primary safety outcome was any serious adverse event during the treatment period. All efficacy and safety analyses were by intention to treat.

**Results:**

4176 patients were assigned to the colchicine group and 4167 were assigned to the placebo group. Stroke occurred within 90 days in 264 patients (6.3%) in the colchicine group and 270 patients (6.5%) in the placebo group (hazard ratio 0.98 (95% confidence interval 0.83 to 1.16); P=0.79). Any serious adverse event was observed in 91 (2.2%) patients in the colchicine group and 88 (2.1%) in the placebo group (P=0.83).

**Conclusions:**

The study did not provide evidence that low-dose colchicine could reduce the risk of subsequent stroke within 90 days as compared with placebo among patients with acute non-cardioembolic minor-to-moderate ischaemic stroke or transient ischaemic attack and a high sensitivity C-reactive protein ≥2 mg/L.

**Trial registration:**

ClinicalTrials.gov, NCT05439356.

## Introduction

Patients with an acute minor-to-moderate ischaemic stroke or transient ischaemic attack remain at considerable risk of subsequent stroke, particularly within the first few days.^1^ ^2^ ^3^ ^4^ An estimate suggests that two thirds of recurrences of stroke within three months occurred within seven days of symptom onset.^5^ ^6^ More importantly, the three month high risk period accounts for approximately 70% of subsequent strokes within one year and 40% within five years.^7^ ^8^ Exploration of novel treatments are needed to reduce the early recurrent risk of stroke and the overall burden of stroke.

Patients with increased inflammation in the acute phase had even higher risk of a new stroke after ischaemic stroke or transient ischaemic attack,^9^ ^10^ ^11^ ^12^ suggesting the potential implications of early anti-inflammatory treatment for reducing the residual recurrent risk of stroke.^13^ ^14^ Colchicine has broad anti-inflammatory effects.^15^ ^16^ ^17^ The Colchicine Cardiovascular Outcomes Trial (COLCOT) found that colchicine reduced the risk of cardiovascular events and stroke as one component of primary endpoints in patients with acute coronary artery disease,^18^ especially in patients who had treatment initiation within three days of myocardial infarction onset.^19^ Together with other secondary prevention trials in cardiovascular disease, collective evidence suggests that colchicine slowed the progression of atherosclerosis by limiting plaque growth, reducing the risk of plaque instability and the risk of in-stent restenosis.^20^ ^21^ The subsequent meta-analyses of the trials involving colchicine has shown that the risk of stroke was almost halved in patients treated with colchicine who had coronary artery disease.^22^ ^23^ ^24^ However, whether colchicine can prevent early subsequent stroke in patients with acute stroke or transient ischaemic attack is still unknown.

In this trial, we aimed to evaluate the efficacy and safety of low dose colchicine versus placebo initiating within 24 h of symptom onset on reducing subsequent stroke within three months in patients with acute non-cardioembolic minor-to-moderate ischaemic stroke or transient ischaemic attack and who have concentration of at least 2 mg/L of high sensitivity C-reactive protein.^25^

## Methods

### Study design

CHANCE-3 (colchicine in high risk patients with acute minor-to-moderate ischaemic stroke or transient ischaemic attack) was an investigator initiated, randomised, double blind, placebo controlled trial conducted at 244 centres in China. The trial rationale, design, and methods have been described in the trial protocol.^26^ Full details of the protocol, statistical analysis plan, committees, sites, investigators, and definitions regarding acute infection, endpoints, and subgroups, such as symptomatic intracranial artery stenosis and symptomatic extracranial artery stenosis,^27^ ^28^ are available in the appendix.

The study was approved by the ethics committee at Beijing Tiantan Hospital (No. KY2022-093-02) and each participating site. Written informed consent was provided by all patients or their representatives before enrolment. The trial was overseen by a trial steering committee. An independent data and safety monitoring committee monitored the progress of the trial, with regular assessment of cumulative safety data to safeguard the wellbeing of the patients.

### Participants

Participants were eligible if they were 40 years of age or older; had either an acute minor-to-moderate ischaemic stroke with a National Institutes of Health Stroke Scale score of 5 or less (range 0-42, with higher scores indicating more severe stroke)^29^ or a high risk transient ischaemic attack with a score of 4 or higher on the ABCD^2^ scale (stroke risk score based on age, blood pressure, clinical features, duration of transient ischaemic attack, and the presence or absence of diabetes mellitus; range 0-7, with higher scores indicating higher risk of stroke);^7^ ^30^ had a high sensitivity C-reactive protein level of ≥2 mg/L at baseline; and could start the trial drug within 24 h from the time at which the patient’s condition was last reported to be normal.

Patients were not eligible if they had a presumed cardiac source of embolus based on medical history, including but not limited to, atrial fibrillation and prosthetic cardiac valve and electrocardiograph at baseline; inflammatory bowel disease or chronic diarrhoea; symptomatic peripheral neuropathy or pre-existing progressive neuromuscular disease; or a non-transient creatine kinase level that was greater than three times the upper limit of the normal range, creatinine level exceeding 1.5 times of the upper limit of normal range, or estimated glomerular filtration rate less than 50 mL/min; severe hepatic disease; clinically significant non-transient haematological abnormalities; acute infection, including respiratory tract infection, urinary tract infection, or gastro-enteritis, or currently using or planning to receive oral or intravenous anti-infective treatment for any other infection; current or planned long term use of systemic glucocorticoid treatment; or planned use of medications that may interact with colchicine. Additional information on inclusion and exclusion criteria is provided in the appendix.

### Randomisation and masking

Eligible participants were randomly assigned in a 1:1 ratio with a block length of four to receive colchicine or placebo colchicine. The randomisation code list, which the trial drug was packaged in accordance with, was generated centrally by a contract research organisation (appendix page 1). The treatment number was allocated using a centralised treatment allocation system at randomisation.

Masking included removing the manufacturer’s label and replacing it with the clinical trial label and randomisation number. Apart from the randomisation number, the pack label text was identical for colchicine and placebo. Patients, care givers, and those assessing outcomes were masked to allocation.

### Procedures

The treatment was initiated within 24 h of symptom onset. Patients randomly received colchicine at a dose of 0.5 mg twice daily or placebo on days 1-3, followed by 0.5 mg per day on days 4 to 90 on a background of standard treatment. Standard treatment included, but was not limited to, antiplatelet drugs, lipid lowering treatment, and control of hypertension and diabetes as applicable at the discretion of the treating physician, according to Chinese Stroke Association guideline for clinical management of cerebrovascular disorders.^31^

Study visits collected outcome data at discharge from the randomising hospital, and at day 90 (plus or minus seven days) via face-to-face interview at each site by trained and certified evaluators who were unaware of the trial group assignments. The exact number of tablets of study drug taken was also recorded at each visit for evaluating treatment adherence.

### Outcomes

The primary efficacy outcome was any new stroke (ischaemic or haemorrhagic) within 90 days after randomisation. Secondary outcomes at 90 days were a vascular event (a composite of stroke, transient ischaemic attack, myocardial infarction, or vascular death), ischaemic stroke, stroke, or transient ischaemic attack, incidence of modified Rankin scale of more than 1 (ranges from 0 to 6, 0 indicating no symptoms; 5 indicating severe disability; and 6 indicating death),^3^ ^32^ and the severity of any recurrent stroke or transient ischaemic attack by means of a six level ordinal scale that combined stroke or transient ischaemic attack events with a score on modified Rankin scale as follows^4^ ^33^: fatal stroke (with subsequent score on the modified Rankin scale of 6), severe stroke (with subsequent score of 4 or 5), moderate stroke (with subsequent score of 2 or 3), mild stroke (with subsequent score of 0 or 1), transient ischaemic attack, and no stroke or transient ischaemic attack.

The primary safety outcome was any serious adverse event within 90 days. Secondary safety outcomes were any adverse event within 90 days and adverse events of special interest as described in the protocol.

The endpoint events were adjudicated by an independent clinical event adjudication committee whose members were unaware of the trial group assignments.

### Statistical analysis

We determined that a total of 8238 patients would provide 90% statistical power to detect a 25% reduction in the risk of any new stroke, with a significance level (α) of 0.05, assuming a 7.1% event risk of the primary outcome in the control group.^1^ ^11^ ^34^

All efficacy analyses were based on the intention-to-treat principle and included all the patients who underwent randomisation. All patients in the intention-to-treat set received at least one dose of the study drug, therefore, the safety analyses were also conducted in this dataset. The crude cumulative incidence of the primary and secondary outcomes was estimated using the Kaplan-Meier method. When multiple events of the same type occurred, the time to the first event was used. Patients without a primary outcome were censored at the time of death, last known contact, or at 90 days, whichever occurred first. The heterogeneity of the treatment effect across the trial centre was visually verified using the forest plot by the trial centres. We compared the difference in event risk between the two trial groups by a shared frailty model, which incorporated the random effect of the trial centre on the baseline hazard of Cox proportional-hazards model^35^ and were reported by hazard ratios and 95% confidence intervals (95% CIs). Proportional hazard assumptions were assessed using log-cumulative hazard plots and by incorporating an interaction term of treatment and time in the Cox model. Absolute event risks and subdistribution hazard ratios from the Fine-Gray mode, which accounts for competing risks of death, were also calculated. For functional outcomes, the incidence of modified Rankin scale scores of more than 1 was reported, generalised linear mixed models were fitted, and an odds ratio was reported. A shift analysis was conducted using ordinal logistic regression and proportional odds assumption was assessed by the χ^2^ score test. The common odds ratio and 95% CI were calculated for the severity of stroke or transient ischaemic attack outcome events measured by the ordinal scale.^4^ ^33^ The common odds ratio was for a one step shift towards the outcome of incidence of ordinal stroke or transient ischaemic attack in the colchicine group compared with the placebo group. All analyses of secondary efficacy outcomes were not adjusted for multiple comparisons and were considered exploratory. Analysis of efficacy outcomes were also performed among the per-protocol set using the same strategy. Prespecified subgroup analyses were done using the shared frailty models as previously specified. The interaction effect between treatment and subgroup variables was tested. We also examined whether any treatment effect differed with age using a continuous scale of age. Safety analysis was conducted by χ^2^ or Fisher’s exact test. A crude two sided P<0.05 was considered as statistical significance. Statistical analyses were done using SAS software, version 9.4 (SAS Institute).

An interim analysis for efficacy was initially planned to be conducted when 60% of patients completed their 90 day follow-up.^26^ However, both steering committee and the independent data and safety monitoring committee recommended to waive the interim analysis because of the high recruitment rate of the trial and because no safety concerned determined by the data and safety monitoring committee .

### Patient and public involvement

The China Guidelines for patient involvement was not available till 21 November 2022 by China Center for Drug Evaluation,^36^ approximately three months after the start of patient enrolment of the CHANCE-3 trial. Patient and public were not involved in design and conduct of this study. The study protocol and manuscript have been widely discussed among physicians and neurologists, who will be involved in disseminating the study findings to healthcare professionals, patients, and members of the public.

## Results

### Patient

**Fig 1:**
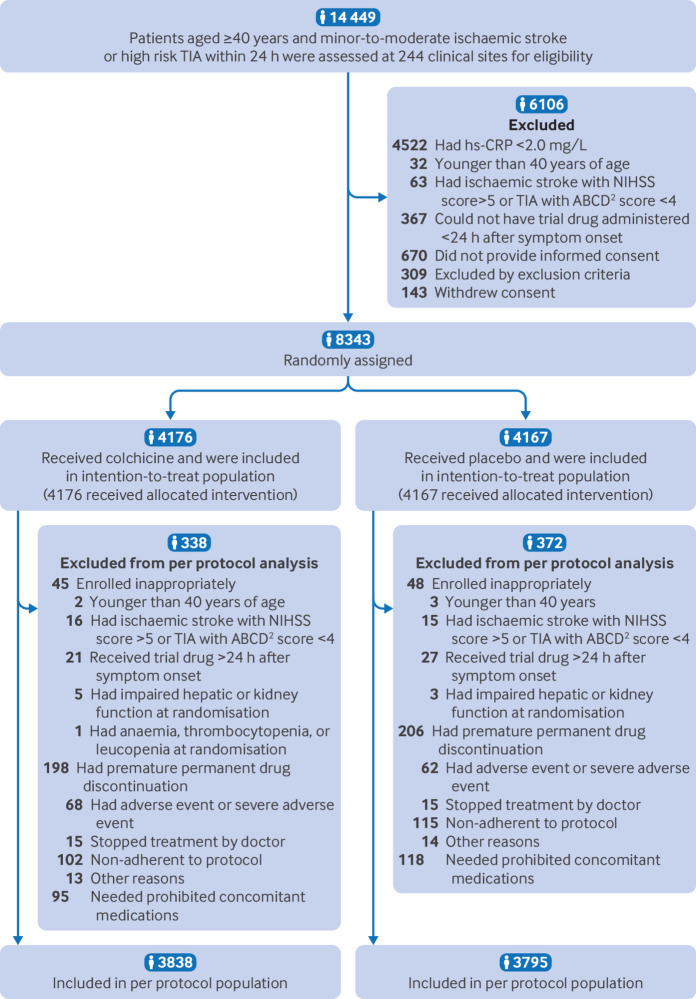
Enrolment and randomisation of the patients. Scores on the National Institutes of Health Stroke Scale (NIHSS) range from 0 to 42, with higher scores indicating more severe stroke. The ABCD^2^ score assesses the risk of stroke on the basis of age, blood pressure, clinical features, duration of transient ischaemic attack, and the presence or absence of diabetes mellitus, with scores ranging from 0 to 7 and higher scores indicating greater risk. hs-CRP=high sensitivity C-reactive protein; TIA=transient ischaemic stroke

Between 11 August 2022 and 13 April 2023, 14 449 patients with ischaemic stroke or transient ischaemic attack were screened at 244 clinical sites; 8343 patients (57.7%) were enrolled, with 4176 randomly assigned to receive colchicine and 4167 receive placebo(fig 1 and table S1 in the appendix, page 21). During the trial, 198 (4.7%) individuals in the colchicine group and 206 (4.9%) participants in the placebo group permanently discontinued their study treatments. All patients who underwent randomisation completed the 90 day (with time window of seven days) follow-up. The primary outcome was available for all patients, including individuals who discontinued their study treatments (fig 1).

Baseline patient characteristics were similar between the two treatment groups (table 1). The median age of the patients was 66.3 years, and 37.6% were women. 7411 patients (88.8%) presented with an ischaemic stroke, and 932 (11.2%) presented with a transient ischaemic attack. The median level of high sensitivity C-reactive protein was 4.8 mg/L. The median time from symptom onset to randomisation was 14.6 h.

**Table 1:** Characteristics of the patients at baseline

Characteristic	Colchicine (n=4176)	Placebo (n=4167)
Age, years	66.3 (58.2-73.1)	66.4 (58.3-73.2)
Sex:		
Male	2632 (63.0)	2578 (61.9)
Female	1544 (37.0)	1589 (38.1)
Body mass index*	24.8 (22.9-27.0)	24.8 (22.9-27.1)
Han Chinese ethnic group†	4056 (97.1)	4047 (97.1)
Comorbidities:		
Hypertension	3175 (76.0)	3223 (77.3)
Diabetes	1331 (31.9)	1346 (32.3)
Dyslipidaemia	1380 (33.0)	1384 (33.2)
Myocardial infarction	57 (1.4)	65 (1.6)
Previous stroke	1194 (28.6)	1219 (29.3)
Current smoker	1051 (25.2)	979 (23.5)
Hs-CRP, mg/L	4.9 (3.0-9.8)	4.7 (2.9-9.3)
Blood pressure, mm Hg:		
Systolic blood pressure	150.5 (138.5-165.5)	150.5 (138.5-166.0)
Diastolic blood pressure	87.5 (80.0-96.5)	87.5 (80.0-96.0)
Time from symptom onset to randomisation, h	14.8 (8.9-20.7)	14.5 (9.2-20.5)
Qualifying event:		
Ischaemic stroke	3718 (89.0)	3693 (88.6)
TIA	458 (11.0)	474 (11.4)
NIHSS score in patients with qualifying ischaemic stroke‡	2.0 (1.0-4.0)	2.0 (1.0-4.0)
ABCD^2^ score in patients with qualifying TIA§	5.0 (4.0-5.0)	5.0 (4.0-5.0)
Previous antiplatelet treatment¶	443 (10.6)	475 (11.4)
Previous lipid-lowering treatment¶	384 (9.2)	412 (9.9)
Symptomatic intracranial artery stenosis	1347/3782(35.6)	1348/3785 (35.6)
Symptomatic extracranial artery stenosis	210/3425 (6.1)	203/3407 (6.0)
Intravenous thrombolysis	524 (12.6)	527 (12.7)
Endovascular thrombectomy	17 (0.4)	17 (0.4)
Medicine use at 90 day follow-up:		
Antiplatelet	4000 (95.8)	3959 (95.0)
Dual antiplatelet treatment	1988 (47.6)	1958 (47.0)
Statin	3988 (95.5)	3976 (95.4)
Other lipid lowering drugs	202 (4.8)	202 (4.8)
Hypoglycaemic drugs**	1000/1331 (75.1)	1034/1346 (76.8)
Antihypertensive drugs††	2336/3175 (73.6)	2329/3223 (72.3)

Data are median (interquartile range), n (%), or n/N (%).

Hs-CRP=high sensitivity C-reactive protein; TIA=transient ischaemic attack; NIHSS=National Institutes of Health Stroke scale.

* BMI is the weight in kilograms divided by the square of the height in metres.

† Ethnic group was reported by the patient and verified by identification card.

‡ NIHSS scores range from 0 to 42, with higher scores indicating more severe neurological deficits. §The ABCD^2^ score assesses the risk of stroke on the basis of age, blood pressure, clinical features, duration of TIA, and the presence or absence of diabetes mellitus, with scores ranging from 0 to 7 and higher scores indicating greater risk.

¶ Patients received medication within one month before symptom onset.

** No of patients with diabetes were 1331 in the colchicine group and 1346 in the placebo group.

†† No of patients with hypertension were 3175 in the colchicine group and 3223 in the placebo group.

The usages of secondary prevention medications (table 1 and table S2, appendix page 23) and prohibited medications during the trial (table S3, appendix pages 24-25) were also similar between the two trial groups.

### Primary and secondary outcomes

**Fig 2:**
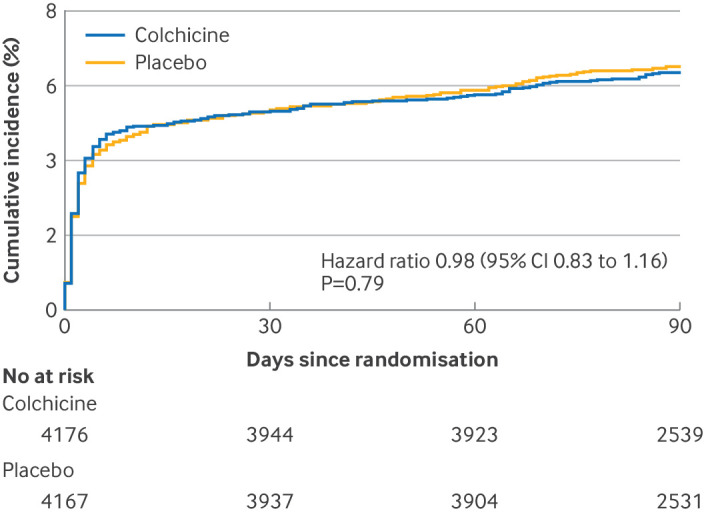
Cumulative incidence of any new stroke (primary outcome) within 90 days. The bottom graph shows the same data on an enlarged Y axis. The event risk and hazard ratio were estimated by Kaplan-Meier method and Cox models without consideration of competing risk of death. P value was calculated from Cox model

A primary outcome event of any new ischaemic or haemorrhagic stroke within 90 days occurred in 264 patients (6.3%) in the colchicine group and in 270 patients (6.5%) in the placebo group (hazard ratio 0.98 (95% CI 0.83 to 1.16); P=0.79) (fig 2 and table 2). Results from the Fine-Gray model, accounting for the competing risk of death, showed similar outcomes (event risk: 6.0% *v* 6.0%; subdistribution hazard ratio 1.00 (95% CI 0.84 to 1.19); P=0.97) (table 2).

**Table 2:** Primary and secondary outcomes at 90 days in patients with minor-to-moderate ischaemic stroke or TIA and a high sensitivity C-reactive protein of at least 2 mg/L assigned to receive colchicine or placebo

Efficacy outcomes	Colchicine (n=4176)			Placebo (n=4167)		Effect type and size (95% CI) *	P value
	No (%)	Event risk*		No (%)	Event risk*		
**Primary outcome**							
Stroke	264 (6.3)	6.3		270 (6.5)	6.5	HR 0.98 (0.83 to 1.16)	0.79
Stroke with competing risk of death		6.0			6.0	SHR 1.00 (0.84 to 1.19)	0.97
**Secondary outcomes**							
Vascular events**†**	298 (7.1)	7.2		309 (7.4)	7.5	HR 0.96 (0.82 to 1.13)	0.64
Vascular events with competing risk of death		7.1			7.4	SHR 0.96 (0.82 to 1.13)	0.62
Ischaemic stroke	257 (6.2)	6.2		263 (6.3)	6.3	HR 0.98 (0.82 to 1.16)	0.79
Ischaemic stroke with competing risk of death		5.9			5.9	SHR 1.00 (0.83 to 1.19)	0.95
Stroke or TIA	284 (6.8)	6.8		290 (7.0)	7.0	HR 0.98 (0.83 to 1.15)	0.79
Stroke or TIA with competing risk of death		6.5			6.5	SHR 1.00 (0.85 to 1.19)	0.98
mRS >1‡	435 (10.4)	—		440 (10.6)	—	OR 0.99 (0.86 to 1.14)	0.86
Ordinal stroke or TIA§						COR 1.03 (0.88 to 1.21)	0.74
Fatal stroke: score of 6 on mRS	12 (0.3)	—		21 (0.5)	—	—	—
Severe stroke: score of 4 or 5 on mRS	44 (1.1)	—		46 (1.1)	—	—	—
Moderate stroke: score of 2 or 3 on mRS	95 (2.3)	—		94 (2.3)	—	—	—
Mild stroke: score of 0 or 1 on mRS	113 (2.7)	—		109 (2.6)	—	—	—
TIA	20 (0.5)	—		20 (0.5)	—	—	—
No stroke or TIA	3892 (93.2)	—		3877 (93.0)	—	—	—

COR=common odds ratio; HR=Hazard ratio; mRS=modified Rankin Scale; OR=odds ratio; SHR=Subdistribution hazard ratio; TIA=transient ischaemic attack..

* Event risks and hazard ratios are estimated by Kaplan-Meier method and Cox models (outcomes without consideration of competing risk of death) or cumulative incidence function and Fine-Gray method (outcomes with consideration of competing risk of death).

† Vascular events were a composite of ischaemic stroke, haemorrhagic stroke, TIA, myocardial infarction, or death from vascular causes.

‡ mRS scores range from 0 to 6, with 0-1 indicating no disability, 2-5 increasing disability, and 6 death.

§ Severity was measured with the use of a six-level ordinal scale that incorporates subsequent stroke or TIA events and the score on the modified Rankin scale at 3 months.

With respect to secondary outcomes, a vascular event occurred in 298 patients (7.1%) in the colchicine group and in 309 patients (7.4%) in the placebo group (hazard ratio 0.96 (95% CI 0.82 to 1.13); P=0.64). Ischaemic stroke occurred in 257 patients (6.2%) in the colchicine group and in 263 patients (6.3%) in the placebo group. A total of 284 patients (6.8%) had stroke or transient ischaemic attack in the colchicine group and 290 (7.0%) in the placebo group. Sensitivity analyses by accounting for competing risk of death showed similar results (table 2). At 90 day follow-up, 435 patients (10.4%) had modified Rankin scale >1 in the colchicine group and 440 patients (10.6%) in the placebo group (odds ratio 0.99 (95% CI 0.86 to 1.14); P=0.86, table 2 and table S4 in appendix page 26). No difference was noted in the incidence and severity of stroke or transient ischaemic attack (using the ordered categorical scale) between two treatment groups (common odds ratio 1.03 (95% CI 0.88 to 1.21), P=0.74; table 2).

**Fig 3:**
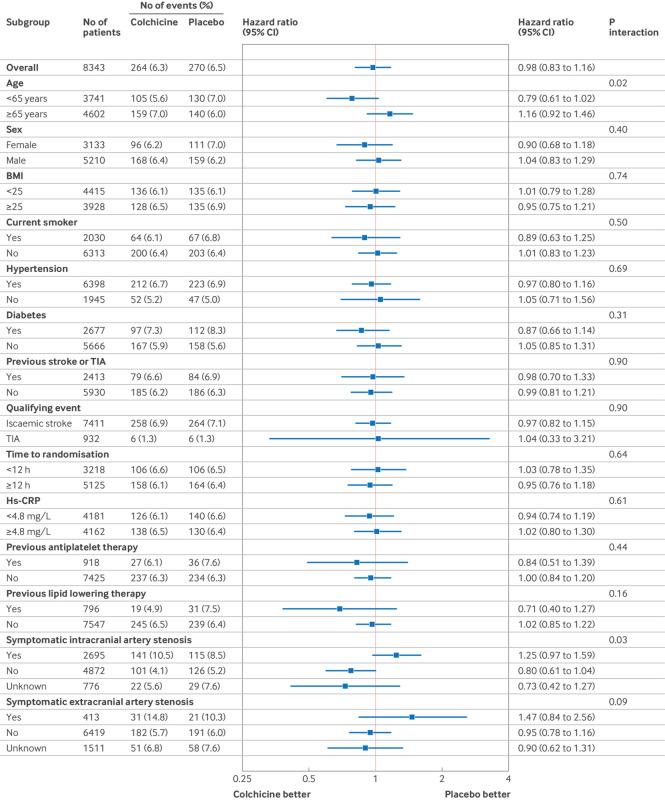
Hazard ratio for any new stroke in prespecified subgroups within 90 days. The body mass index is the weight in kilograms divided by the square of the height in metres

When the primary outcome was assessed in prespecified subgroups, treatment effects might be different between the two trial groups according to ages (crude interaction P=0.02 when age is dichotomised, and P=0.018 when age is treated as a continuous variable) (fig 3, figure S1 in appendix page 36), and among the three levels of the status for symptomatic intracranial artery stenosis (crude interaction P=0.03) (fig 3). We did not find any potential heterogenetic treatment effect of colchicine in other prespecified subgroups (fig 3). The forest plot by the trial centres justified using shared frailty models to address treatment effect heterogeneity across centres (figure S2 in appendix page 37).

The results of the per protocol analysis were similar to those of the primary intention-to-treat analysis (table S5, appendix pages 27-28).

### Safety and adverse events

Serious adverse events occurred in 91 (2.2%) patients in the colchicine group and in 88 (2.1%) patients in the placebo group (table 3). Fifteen patients (0.4%) in the colchicine group and 18 patients (0.4%) in the placebo group died from non-cardiovascular causes. During the trial, 910 patients (21.8%) had any adverse event in the colchicine group and 888 (21.3%) in the placebo group (table 3).

**Table 3:** Adverse events and serious adverse events within 90 days in patients with minor-to-moderate ischaemic stroke or transient ischaemic attack and a high sensitivity C reactive protein of at least 2 mg/L assigned to receive colchicine or placebo

Safety outcomes	Colchicine (n=4176)	Placebo (n=4167)	P value
**Primary safety outcome**			
Any serious adverse event	91 (2.2)	88 (2.1)	0.83
**Other safety outcomes**			
Serious adverse event:			
Death	34 (0.8)	45 (1.1)	0.21
Cardiovascular death	19 (0.5)	27 (0.7)	0.23
Non-cardiovascular death	15 (0.4)	18 (0.4)	0.60
Gastrointestinal event	8 (0.2)	7 (0.2)	0.80
Infection	4 (0.1)	5 (0.1)	0.75*
Pneumonia	16 (0.4)	7 (0.2)	0.06
Adverse events:	910 (21.8)	888 (21.3)	0.59
Gastrointestinal event	173 (4.1)	150 (3.6)	0.20
Diarrhoea	71 (1.7)	30 (0.7)	<0.001
Flatulence	21 (0.5)	10 (0.2)	0.05
Constipation	30 (0.7)	45 (1.1)	0.08
Dyspepsia	23 (0.6)	29 (0.7)	0.40
Gastrointestinal haemorrhage	9 (0.2)	15 (0.4)	0.22
Others	25 (0.6)	26 (0.6)	0.88
Anaemia	27 (0.6)	20 (0.5)	0.31
Leukopenia	5 (0.1)	0 (0.0)	0.06*
Thrombocytopenia	8 (0.2)	3 (0.1)	0.23*
Myopathy	0	0	
Increased CK levels†	0	0	
Increased ALT or AST levels	29 (0.7)	19 (0.5)	0.15
Abnormal hepatic function‡	12 (0.3)	3 (0.1)	0.03*

Data are number (percentage). CK=creatine kinase; ALT=alanine transaminase; AST=aspertate aminotransferase.

* P value was calculated by Fisher’s exact test.

† At least five times the upper limit of normal.

‡ ALT or AST at least three times the upper limit of the normal range.

Despite the use of statins within 90 days in 7964 (95.5%) patients, no myopathy was reported in both trial groups (table 3 and table S6, appendix page 29). Twelve (0.3%) patients had abnormal hepatic function within 90 days in the colchicine group compared with three (0.1%) in the placebo group (P=0.03; table 3 and table S6, appendix page 29). Diarrhoea occurred in 71 patients (1.7%; six patients had a stroke within 90 day) in the colchicine group and in 30 patients (0.7%; one patient had a stroke within 90 day) in the placebo group (P<0.001; table 3 and table S6, appendix page 29). The risk of serious adverse events and adverse events classified by system organ class were similar between the two trial groups (table S7, appendix page 30, and table S8, appendix page 31).

Sixty eight patients in the colchicine group (1.6%) and 62 patients in the placebo group (1.5%) prematurely discontinued study treatments owing to serious adverse events or adverse events (table S9, appendix page 32). The rates of use of any or dual antiplatelet treatment, statin, hypoglycaemic, and antihypertensive drugs within 90 days among patients with serious adverse events, adverse events, or premature discontinuation of study treatments, were similar between two trial groups (table S10-12, appendix pages 33-35).

## Discussion

### Principal findings

In this randomised, double blind, placebo controlled trial in participants with acute minor-to-moderate ischaemic stroke or high risk transient ischaemic attack and a high sensitivity C-reactive protein concentration of at least 2 mg/L at baseline, low dose colchicine initiated within 24 h of symptom onset was not superior to placebo in reducing the risk of subsequent stroke within 90 days.

### Comparison with other studies

Our previous study of patients with acute ischaemic stroke from independent cohorts showed that risk of recurrent stroke increased by continuous increase in high sensitivity C-reactive protein concentration or by use of a cut-off of 2 mg/L at baseline.^11^ ^25^ Moreover, the association of high sensitivity C-reactive protein with recurrence of stroke was observed when this protein was tested within 24 h, but the association disappeared if detecting concentrations of the protein between 72 h and 8 days.^37^ The measurement of high sensitivity C-reactive protein is common in clinics and its concentration can usually be obtained rapidly. The median time from symptom onset to obtaining test results for this protein was 10.4 h in CHANCE-3, suggesting no delay for initiating the trial drugs caused by the assay. Taken together, patients who were at high risk were intended to be recruited for anti-inflammatory treatment by using a high sensitivity C-reactive protein cut-off of 2 mg/L. However, the necessity of this measurement for screening patients still needs further investigation.

Use of low-dose colchicine as an anti-inflammatory drug resulted in lower cardiovascular event rates over a median of 22.6 months in the COLCOT trial, which included patients in an acute condition after myocardial infarction and had completed planned percutaneous revascularisation procedures.^18^ ^19^ Our results differ from that of the COLCOT trial and various reasons might explain these differential findings. Of note, the purpose of our study was not identical to COLCOT. As the time course analyses of the CHANCE-2 and POINT trials showed, which was also indicated in this study, most recurrences occurred within seven days of symptom onset.^5^ ^6^ We focused on this highest risk period for stroke and tested the effect of colchicine on preventing very early recurrent stroke. The 90 day duration of colchicine treatment in CHANCE-3 was thus different from COLCOT,^18^ ^19^ which concentrated on long term risk of cardiovascular events. Similarly, another ongoing phase 3 randomised trial of CONVINCE (Colchicine for prevention of vascular inflammation in non-cardioembolic stroke), which included patients 72 h after ischaemic stroke and transient ischaemic attack, aims to determine the long term effectiveness of colchicine as a secondary prevention strategy.^38^ In addition, important pathophysiological differences exist in early recurrent events between coronary heart disease and ischaemic stroke.^39^ ^40^ How inflammation is involved in early prognosis of acute ischaemic stroke and high risk transient ischaemic attack is unclear. Therefore, low-dose colchicine may not be the appropriate drug for the short term secondary preventive treatment in this study population. Premature permanent discontinuation of colchicine might influence the evaluation of its treatment effect. In the per protocol analysis, however, the conclusion of non-effectiveness of colchicine was sustained after removal of the patients with premature discontinuation of study drugs from the analysis. We found that six of 71 patients (with diarrhoea) in colchicine group and one of 30 (with diarrhoea) in the control group had an event of stroke during the trial. Such a small difference could not explain the findings in this trial.

A meta-analysis of 15 randomised controlled trials in patients with coronary heart disease showed that colchicine significantly reduced the risk of cardiovascular events in patients up to 65 years of age, but not in those older than 65 years.^24^ In this study, we observed a similar trend in patients younger than 65 years old as compared with that in individuals older than 65 years. Systemic inflammation increased with the advancement of age.^41^ Inflammation in older adults might be more complex with more contributing factors, such as atherosclerosis, infection, and comorbidity. Whether colchicine affected a specific factor and what that was is unclear, as is the different inflammatory pathways that might be involved in cerebrovascular ageing. In the subgroup analyses, crude P values were reported for treatment by prespecified factor interaction effect individually without adjustment for multiple testing. Therefore, we could not rule out the possibility of chance findings. In addition, all the subgroup analyses were exploratory in nature, and these findings warrant further investigation.

We found no evidence of safety concerns on serious adverse events with colchicine during the trial. Consistent with previous colchicine trials,^18^ ^42^ a difference was noted in the rates of diarrhoea, flatulence, and abnormal hepatic function between patients receiving colchicine and those receiving placebo. However, the overall incidences were lower in this study population, partially due to short duration of treatment.

### Limitations

The CHANCE-3 trial has some limitations. In this study, we tested only high sensitivity C-reactive protein at baseline to screen patients and did not collect patients’ biosamples at their 90 day follow up. Therefore, we were not able to evaluate the biological effects of low-dose colchicine during the 90 day treatment period. The absence of follow up of concentrations for high sensitivity C-reactive protein also made the interpretation of the results challenging. The benefit of long term treatment of colchicine in reducing the risk of stroke were shown to be higher than that of short term treatment in patients with coronary disease.^24^ Our findings in this study are from a 90 day treatment period, which was relatively short, and therefore did not imply that a long term duration of colchicine would not be beneficial. Additionally, the 95% CI of the hazard ratio for the primary efficacy outcome contained clinically relevant values, especially the lower limit of 0.83, and uncertainty still exists as to the treatment effect. It warrants further validation studies. In this study, the general categories of stroke secondary prevention medication were available without collecting their exact drug types, such as antihypertensive and antidiabetic drugs. Therefore, we were not able to access whether any drugs used for secondary prevention might influence the colchicine effect. Furthermore, the relative short time window for enrolment in the study limited routine assessment of cardio-embolism, including 24 h Holter electrocardiograph monitoring, and evaluations to rule out alternative causes for strokes before randomisation. We did not collect the data for these assessments during the study period either, which may provide better insights into the absence of colchicine effect. Although commonly seen in previous secondary stroke prevention trials, women were under-represented in this study. To promote equal sex representation, stratified sampling design may be considered in future studies. In addition, this study recruited patients from 11 August 2022 and completed recruiting on 13 April 2023. Fewer patients were included in summer, which might also limit the interpretation of the trial’s findings. Both intracranial and extracranial atherosclerotic diseases are known risk factors for recurrent ischaemic stroke.^43^ ^44^ In Asian populations, the incidence of intracranial atherosclerosis is generally high without features of complicated plaques.^45^ ^46^ ^47^ ^48^ We did not collect the data regarding intracranial and extracranial atherosclerosis or histology of either the index events or the new stroke events at 90 day follow-up. We, therefore, were not able to evaluate atherosclerotic situations and its potential impact on the effect of colchicine for the patients participated in this study. Due to lack of information for colchicine treatment in patients with stroke in the literature, we adopted a suboptimal approach to calculate the sample size for this study as described previously,^26^ which might lead to either an under or over powered study. Our findings of this study may not be generalisable to populations other than Asian patients.

### Conclusions

This trial of participants with non-cardioembolic minor-to-moderate ischaemic stroke or high risk transient ischaemic attack and with a baseline concentration for high sensitivity C-reactive protein of at least 2 mg/L, did not provide sufficient evidence that low-dose colchicine initiated within 24 h of symptom onset could reduce the risk of subsequent stroke or increase the risk of serious adverse events as compared with placebo within 90 days.

**What is already known on this topic**

Inflammation has been associated with incidence and recurrence of stroke, and risk of stroke was reduced in patients who have coronary artery disease and who were treated with colchicine
However, evidence is lacking regarding efficacy and safety of acute use of colchicine for the prevention of early recurrent stroke

**What this study adds**

No differences were noted in treatment effects on subsequent stroke between the low dose colchicine and the placebo groups
Additionally, no difference was noted in the primary safety outcome for any serious adverse event

## Data Availability

Data collected for the study, including de-identified individual participant data and a data dictionary defining each field in the set, can be made available to other researchers on reasonable request and after signing appropriate data sharing agreements. Please send data access requests to the corresponding author. Such requests must be approved by the respective ethics boards and appropriate data custodians.

## Web extra

Extra material supplied by authors

Web appendix: Extra material supplied by authors

## Notes

Contributors: A full list of the CHANCE-3 investigators is provided in the appendix. JL, XM, F-DS, and JJ contributed equally to the manuscript. JL, XM, F-DS, JJ, and YW prepared the first draft of the report. HL, F-DS, SCJ, GJH, DE, YW, XZ, ZL, LL, and HZ conceptualised the study design and provided critical comments for the manuscript. YJ and WY managed the data. HG, AW, and YP calculated the sample size and developed the statistical plan. H-QG and AJ did the statistical analyses, with supervision by HL. All other authors were local investigators or co-investigators and recruited participants, collected data, revised the final version of the manuscript, and critically reviewed the report and approved the final version before submission. The steering committee was responsible for the overall design, protocol development, interpretation, and supervision of the trial. The trial executive committee implemented the study. The corresponding author acted as the guarantor for the study, had full access to all the data in the study, had final responsibility for the decision to submit for publication, and attested that all listed authors meet authorship criteria and that no others meeting the criteria have been omitted.

Transparency: The corresponding author affirm that the manuscript is an honest, accurate, and transparent account of the study being reported; that no important aspects of the study have been omitted; and that any discrepancies from the study as planned and registered have been explained.

Dissemination to participants and related patient and public communities: The public media and clinical sites will report the findings of CHANCE-3 and provide the text link to the audiences and patients.

Provenance and peer review: Not commissioned; externally peer reviewed.
