# Supplementary material for: Colchicine in patients with acute ischaemic stroke or transient ischaemic attack (CHANCE-3): multicentre, double blind, randomised, placebo controlled trial
Source: BMJ. 2024 Jun 26;385:e079061. doi: 10.1136/bmj-2023-079061 (PMC11200154; doi:10.1136/bmj-2023-079061)

Supplementary Appendix

Supplement to: Colchicine in Patients with Acute Ischaemic Stroke or Transient Ischaemic Attack

Listing of committees and coordinating centres in the CHANCE-3 trial	Page 1
Listing of participating sites and investigators in the CHANCE-3 trial	Page 2
Study design and treatment allocation of the CHANCE-3 trial	Page 11
Inclusion and exclusion criteria of the CHANCE-3 trial	Page 12
Definition of acute infection	Page 15
Definition of stroke events and vascular events	Page 16
Definition of symptomatic intracranial artery stenosis and symptomatic extracranial artery stenosis	Page 19
Supplementary Tables S1-S12	Page 21
Supplementary Figure S1-S2	Page 36

Listing of committees and coordinating centres in the CHANCE-3 trial

Steering Committee: Yongjun Wang MD., S. Claiborne Johnston MD, PhD., Graeme J Hankey MD, J. Donald Easton MD, Fu-Dong Shi MD, PhD, and investigators from the participating hospitals.

Executive Committee: Yongjun Wang MD., Hao Li MD, PhD., Xia Meng MD, PhD., Jiejie Li MD, PhD., Jing Jing MD, PhD., Xuewei Xie MD, PhD. Jinxi Lin MD, PhD.

Data and Safety Monitoring Board: Anding Xu MD, PhD., Hui Zhi MD, PhD., Qiang Dong MD, PhD., Dongsheng Fan MD, PhD., Bin Peng MD, PhD.

Clinical Event Adjudication Committee: David Wang D.O., FAAN, FAHA, James Wang M.D.FAAN, Jindong Xu M.D.

Clinical Coordinating Centre: Xia Meng MD, PhD., Jiejie Li MD, PhD., Jing Jing MD, PhD., Anxin Wang MD, PhD., Xuewei Xie MD, PhD., Jinxi Lin MD, PhD., Siying Niu MD.

Data Management and Statistics Center: Hao Li MD, PhD., Yong Jiang MD, PhD., Hongqiu Gu MD, PhD., Aoming Jin MD, PhD., Weiran Yu MS.

Drug distribution centre: Bin Li (CRO)

Independent medical monitor: Wei Shi, Bin Li (CRO)

Listing of participating sites and investigators in the CHANCE-3 trial

Beijing Tiantan Hospital, Capital Medical University Xingquan Zhao

Liaocheng Third People's Hospital Liguo Chang

Jingdezhen No.1 People's Hospital Minghua Cao

Yanggu Traditional Chinese Medical Hospital Yanliang Miao

Mengzhou People’s Hospital (Department of Neurology ward 1) Dali Li

Liaocheng people's hospital Xiafeng Yang

Xi 'an Fengcheng Hospital Aimei Wu

Yantai Penglai traditional Chinese medicine hospital Penglai Shi

The First People's Hospital of Lanzhou City Jianghua Si

Jiyuan Hospital of Traditional Chinese Medicine Hongqin Yang

The First Affiliated Hospital of Xiamen University Naian Xiao

Hebei Tang County People Hospital Na Guo

Puding County People's Hospital Ling Ma

Yiyang County People's Hospital Youren Li

Qianxinan Buyi and Miao Autonomous Prefecture People's Hospital Tianming Pan

Qinghe people's hospital Yingzhuo Zang

Yugan Xinjiang hospital Fangqi Tian

Tuoketuo County Hospital Youjun Liu

Xiushan Tujia and Miao Autonomous County People's Hospital Xiaopeng Feng

Hejian People’s Hospital Dongqi Liu

Chongqing Sanbo Chang 'an Hospital Xianwen Han

Nantong Tongzhou District No.8 People's Hospital Yi Zhao

Nanjing Drum Tower Hospital Group Suqian Hospital Xueling Zhang

Inner Mongolia International Mongolian Hospital Mei Hong

Sinopharm North Hospital Airen Lu

The Fourth People's Hospital of Shangqiu Haichao Liu

Luoyang Mengjin District People's Hospital Zhonghai Jia

Handan First Hospital Kai Li

The People's Hospital of the Qiandongnan Miao and Dong Autonomous Prefecture Xiaosong Li

The people’s Hospital of Wuhai Ziyan Li

Beijing Shunyi Airport Hospital Dongli Chen

Tonggu County People's Hospital Lifeng Lei

Neixiang County People's Hospital Jingwen Jiao

Lankao County Central Hospital Baohua Zhao

Dengzhou Central Hospital Peiduo Yuan

Yangxin County People Hospital Xingchi Wang

Wangdu County Hospital,Hebei Province Qiang Li

Xinmi Hospital of T.C.M Jianmin Guo

Qiu County People's Hospital Xin Wang

Werixian People's Hospital Cunrui Wang

Yicheng Municipal Hospital of Zaozhuang Haifeng Huang

Hebi Coal Industry (Group) Co., Ltd. General Hospital Yanhua Zhang

The Second People's Hospital of Huludao Yefang Feng

Baotou City Central Hospital Baojun Wang

Weishi Central Hospital Weifeng Lu

The People's Hospital of Gaotang Huqing Li

Jiangyin People's Hospital Bojun Han

The second Affiliated Hospital of Guizhou Medical University Jianghuan Zheng

The First Hospital of Fangshan District, Beijing Jianhua Li

Zhecheng People's Hospital Hongtian Zhang

Chongqing Tungwah Hospital Yu Che

Ulanpab Central Hospital Yuhai Liu

Boai People's Hospital Jianli Cheng

Central Hospital of Wafangdian Ni Wang

Shijiazhuang Third Hospital Yunshu Zhang

Dalian Lvshun District People's Hospital Dongyun Li

Affiliated Hospital of Inner Mongolia Minzu University Dongwei Zhang

The People’s Hospital of Qihe County Haitao Li

Xichuan County People's Hospital Baolong Wang

Guangdong second Provincial Central Hospital Xintong Liu

People's Hospital of Zhongwei Tianhui He

The Third Hospital of Xiamen Duanling Ye

The Second People's Hospital of Guiyang Ping Sun

The Third People's Hospital of Guiyang Anqiang Chen

The Sanming First Hospital Affiliated to Fujian Medical University Weimin Hong

The People's Hospital of Cenxi City Hongbin Liang

Tongliao City Hospital Yanqiu Du

Weihai Wendeng District People's Hospital Jinguo Zhao

The second people's hospital of Pingdingshan Min Zhang

Xingyang People's Hospital Weifeng Chen

Liaocheng City Central Hospital Xiting Zhang

Linfen Central Hospital(emergency department) Hongguo Dai

Yixing People's Hospital Junfeng Shi

Ye County People's Hospital Ke Li

Siping Central People's Hospital Junfeng Zhao

Xinghua City People's Hospital Qian Wang

Juancheng County People's Hospital Yuqing Zhang

Dalian PuLanDian central hospital Yajun Liu

Panzhihua Central Hospital Xiangming Wang

Guantao county central Hospital Bin Li

People's Hospital of Yinan Bingqi Zhang

Luoning County People's Hospital Xiaomin Mei

YEDA hospital Mingqing Zhen

Nanyang Second General Hospital Jinhui Qin

Fanxian People's Hospital Yanling Hu

Liaocheng People's Hospital Cunju Guo

Xianyang Hospital of Yan 'an University Li Ji

Zibo Municipal Hospital, Zengqiang Sun

The Shangqiu First People's Hospital Yunyi Dai

The Second Affiliated Hospital of Hainan Medical University Yong You

Zhejiang Provincial People's Hospital Bijie Hospital Bo Wang

Dalian lvshunkou district traditional Chinese medicine hospital Changhao Jiang

Yantai Affiliated Hospital of Binzhou Medical University Rong Zou

Zichang People's Hospital Runting Jing

The First Affiliated Hospital of Chongqing Medical and Pharmaceutical College Di Pu

Linyi Third People's Hospital Pida Hao

Guanxian People's Hospital Defeng Tian

The First Affiliated Hospital of Hebei North University Qian Xue

Yantai Yuhuangding Hospital Zhigang Liang

The No.4 People's Hospital of Hengshui (Department of Neurology ward 1) Aisheng Wu

The Hospital of Anguo City Jingya Jiao

The First People Hospital of Lingbao Wei Wu

Shunping County Hospital Dong Wang

Anshan Central Hospital Zhen Jiao

The Second affiliated Hospital of Xiamen Medical College Jianping Niu

Jiujiang University Affiliated Hospital Xiangbin Wu

Inner Mongolia Forestry General Hospital Jing Wang

Shaodong People's Hospital Ping Shen

The people’s Hospital of Wulateqianqi Zhao Li

Huixian People's Hospital Huihai Du

Yunnan Nujiang Lisu Autonomous Prefecture People's Hospital Liqin He

Zhangye People's Hospital Affiliated To Hexi University Xinhui Kou

Taikang Xian People's Hospital Jing Chen

Beijing Shunyi Hospital Quping Ouyang

Linquan County People's Hospital Youquan Ren

Ningjin People's Hospital Chunjie Yang

PKUcare Zibo Hospital Hailian Jin

Wuyuan County People's Hospital Yongming Chen

The Second People's Hospital of Dongying Chunlei Li

East District of The First Affiliated Hospital of Xi'an Jiaotong University Qiuwu Liu

The Sixth People's Hospital of Nantong Hongliang Wang

Zibo Hospital of Traditional Chinese Medicine Hao Hu

Sui county traditional Chinese medicine hospital Ying Li

Qinyang City Hospital of Traditional Chinese Medicine Gongping Zhu

Baise City People's Hospital Shaofa Li

Baoding Xushui District People's Hospital Hongyun Chang

The Third People's Hospital of Chenzhou Huixiang Tang

Puyang County People's Hospital Sumin Bai

Shenqiu County People's Hospital Weidong Jia

Huhhot First Hospital Wensheng Tian

Binzhou Central Hospital Shuzhen Yang

Haiyan People's Hospital Zhenhua Xi

Hongxinglong Hospital of Beidahuang Group Degang Sun

Central Hospital of Yongcheng Can Jiang

The People's Hospital of Rugao Yueqiang Gao

Zhumadian TCM Hospital Xia Wei

The Fourth People's Hospital of Hengshui (Department of Neurology ward 2) Jianling Zhang

The Affiliated Hospital of Xinyang Vocational and Technical College Rongfang Ma

People's Hospital of Anji Fujian Chen

Siyang kangda hospital Yanan Zhu

The second People Hospital of Xinxiang Henan Hongjuan Chang

The First People's Hospital of Wuhu Houqin Chen

Wuzhi County People's Hospital Guoyou Zhao

Ninger County People's Hospital Junyong Zhao

Hengyang Central Hospital Jing Ding

Nanjing Gaochun People's Hospital Shoucheng Zhang

Meihekou Central Hospital Rui Wang

Suxitong Science and Technology Industrial Park People's Hospital Panbing Huang

Yantai Zhifu hospital Jingyuan Jiang

Chenzhou No.1 People‘s Hospital Haipeng Li

The people’s Hospital of Linqing Wei Zhang

The People's Hospital of Anyang City Qingcheng Yang

Guihang Guiyang hospital Chen Niu

Yueyang People's Hospital Ke Deng

Ningde People's Hospital Guoping Zou

Yantai Penglai People's Hospital Zhongfeng Yu

The Fourth People's Hospital of Chenzhou Yongqian Lei

Hengshui People's Hospital Yan Wei

Qiu County Hospital of Traditional Chinese Medicine Tao Sun

The Third Hospital of Mianyang Diwen Zhang

Luoyang Dongfang People's Hospital Hongliang Wang

Qinyang People's Hospital Jiafeng Dong

Yichun People's Hospital Xinbo Deng

Dalian Municipal Central Hospital Dong Chen

Central Hospital of Zhuanghe City Hongyan Ni

Guyuan people's hospital Lujun Zhang

Ruyang People's Hospital Guofeng Li

Linyi Central Hospital Shifeng Guo

The Second Hospital of Hebei Medical University Dan He

The First Affiliated Hospital of Baotou Medical College   Lie Wu

Yuncheng County People's Hospital   Guoqiang Yang

Affiliated Hospital of Liaoning University of Traditional Chinese Medicine   Ying Hai

The First People's Hospital of jiande   Dongjing Song

Yueyang Hospital of Integrated Traditional Chinese and Western Medicine, Shanghai University of Traditional Chinese Medicine   Yan Han

Xihua Xian People's Hospital   Chaoqun Li

Second Hospital of Shanxi Medical University   Li Wang

The Second Affiliated Hospital of Xuzhou Medical University   Xiue Wei

Inner Mongolia People's Hospital   Runxiu Zhu

Fuzhou First People's Hospital of Jiangxi Province   Haiyan Xu

Kaifeng Central Hospital   Xinsheng Han

The Second Affiliated Hospital of Henan University of Science and Technology   Wen Shangguan

ShangRao People's Hospital   Ailian Zhang

Jiangsu Rudong County People's Hospital   Jun Gu

Ruzhou People's Hospital   Peng Guo

Weihai Municipal Hospital   Hairong Sun

Mengzhou Hospital of Traditional Chinese Medicine   Baoguo Xue

The 2nd Affiliated Hospital of Harbin Medical University   Lihua Wang

The Fifth Affiliated Hospital of Jinan University (Heyuan Shenhe People's Hospital) Wanyong Yang

Qingdao Fuwai Cardiovascular Hospital   Shihao You

Chengde County Hospital   Mingjie Liu

Dalian Jinzhou District First People's Hospital   Huijuan Sun

The First Affiliated Hospital of Jinan University   Anding Xu

Sichuan Provincial People's Hospital   Nengwei Yu

The First People's Hospital of Guiyang   Yafei Shangguan

First Affiliated Hospital of Kunming Medical University   Lianmei Zhong

The Second People's Hospital of Liaocheng   Xinqiang Wang

Xinxiang tongmeng hospital   Hejun Chen

Shengjing Hospital of China Medical University   Juan Feng

Affiliated Hospital of Shandong University of Traditional Chinese Medicine Xiangqing Xu

Affiliated Hospital of Chengde Medical University   Xiaoxuan Zhang

Qingfeng xinxing hospital   Jianguo Ge

The First People's Hospital of Longquanyi Disdrict,Chengdu   Meirong Zhu

Ankang Central Hospital   Dongbo Li

The Second People's Hospital of Neijiang   Jiajun Huang

Qixia traditional Chinese medicine hospital   Xiaoyan Li

Luoyang Central Hospital   Zhihui Duan

Affiliated Hospital of Hebei University   Weiying Di

Xixia County People's Hospital   Jianchao Qin

Fuxing Hospital, Capital Medical University   Fang Li

Xiuwu County People's Hospital   Guangming Kang

Chongqing University Fuling Hospital   De Yang

Jiangxi Provincial People's Hospital   Wenfeng Cao

Huizhou First Hospital   Chunsheng Cai

Zibo Central Hospital   Xiangqing Li

Dalian University Affiliated Xinhua Hospital   Yi Wang

Tengchong People's Hospital   Shengfu Yang

Nanfang Hospital，Ganzhou   Xianghong Liu

Jiujiang No.1 People's Hospital   Hebin Wan

The People's Hospital of Liaoning Province   Muhui Lin

Shanghai Seventh People's Hospital   Feng Wang

China-Japan Union Hospital of Jilin University   Ying Xing

Linyi Traditional Chinese Medicine Hospital   Qiangyuan Tian

Linfen Central Hospital (neurology department)   Wanying Li

Weihai Central Hospital　Honghao Man

Yantai Taocun Central Hospital　Zhihua Hao

Linyi People's Hospital　Ziran Wang

Panjin Central Hospital　Yanhua Zhou

Shanxi Bethune Hospital　Xinyi Li

People's Hospital of Deyang City　Hong Chen

People’s Hospital of Xinjiang Uygur Autonomous Region, Urumqi, China Hongyan Li

Ordos central hospital　Junmei Wang

Luoyang First People’s Hospital　Jinfeng Shi

Mengzhou People's Hospital(Department of Neurology ward 2)　Dali Li

The First Affiliated Hospital of Shihezi University　Juan Tang

The First Affiliated Hospital of Harbin Medical University　Di Zhong

North China University of Science And Technology Affiliated Hospital　Bin Liu

Qixia City People's Hospital　Chuanzhen Qu

Tangshan Gongren Hospital　Haiying Wang

Shanxi Cardiovascular Hospital　Chen Chen

Affiliated Zhongshan Hospital of Dalian University　Xiangyu Pu

The First People's Hospital of Foshan　Yukai Wang

The Second Affiliated Hospital of Chongqing Medical University　Yangmei Chen

The First Affiliated Hospital of Wannan Medical College　Yang Xu

Huangshan City People's Hospital　Fei Wang

Study design and treatment allocation

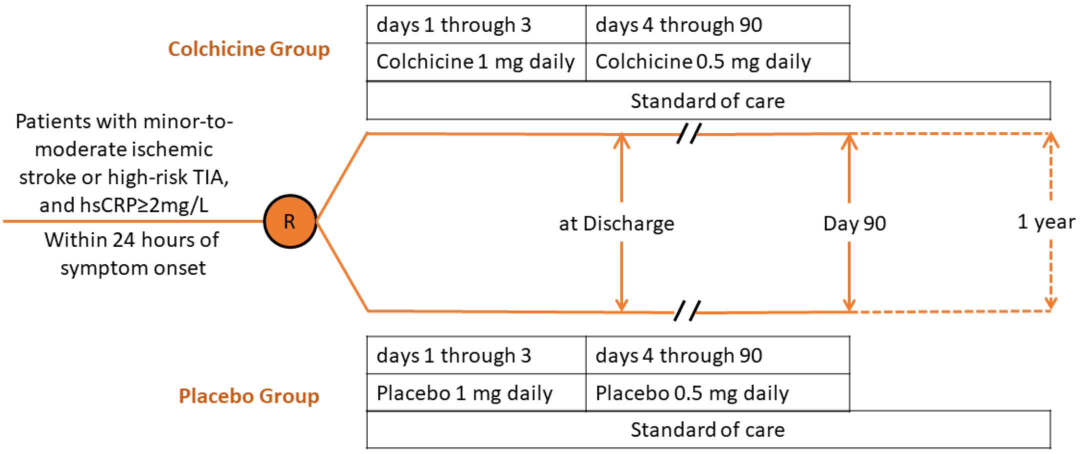

Inclusion and exclusion criteria

Inclusion Criteria

1. ≥40 years old;

2. Acute cerebral ischaemic event due to:

• Acute minor-to-moderate ischaemic stroke (NIHSS≤5 at the time of randomisation) or,

• TIA with moderate-to-high risk of stroke recurrence ($ABCD^2$ score ≥ 4 at the time of randomisation);

1. With a hsCRP level of ≥2mg/L at randomisation;
2. Can be treated with study drugs within 24 hours of symptoms onset*(*Symptom onset is defined by the "last see normal" principle);
3. Informed consent signed.

Exclusion Criteria

1. Malformation, tumor, abscess or other major non-ischaemic brain disease (e.g., multiple sclerosis) on baseline head CT or MRI.
2. Isolated or pure sensory symptoms (e.g., numbness), isolated visual changes, or isolated dizziness/vertigo without evidence of acute infarction on baseline head CT or MRI.
3. Iatrogenic causes (angioplasty or surgery) of stroke or TIA.
4. Presumed cardiac source of embolus, such as atrial fibrillation or prosthetic cardiac valve.
5. A score of ≥ 2 on the modified Rankin scale immediately before the occurrence of the index event.
6. Usage of colchicine within 30 days before randomisation or planning to take colchicine therapy for other indications.
7. Known allergy or sensitivity or intolerance to colchicine.

8. Inflammatory bowel disease (Crohn's or ulcerative colitis) or chronic diarrhea.
9. Symptomatic peripheral neuropathy or pre-existing progressive neuromuscular disease or with creatine kinase (CK) level > 3 times the upper limit of normal as measured within the past 30 days and determined to be non-transient through repeat testing.
10. A history of cirrhosis, chronic active hepatitis or severe hepatic disease.
11. Impaired hepatic (ALT or AST > twice the upper limit of normal range) or kidney (creatinine exceeding 1.5 times of the upper limit of normal range or eGFR less than 50 ml/min) function at randomisation.
12. Anemia (haemoglobin <10g/dL), thrombocytopenia (platelet count <100×109/L) or leucopenia (white blood cell <3×109/L) at randomisation.
13. In the acute phase of respiratory tract infection, urinary tract infection, and gastro-enteritis, or currently using or planning to receive oral or intravenous anti-infective therapy for any other infection.
14. Currently using or planning to begin long-term (>7 days) systemic anti-inflammatory drugs (NSAIDs except for aspirin, oral or intravenous steroid therapy) during the study.
15. Planning to use moderate or strong CYP3A4 inhibitors (clarithromycin, erythromycin, telithromycin, other macrolide antibiotics, ketoconazole, itraconazole, voriconazole, ritonavir, atazanavir, indinavir, other HIV protease inhibitors, verapamil, diltiazem, quinidine, digoxin, disulfiram, etc) or P-gp inhibitors (cyclosporine) at randomisation.
16. Planned surgery or interventional treatment requiring cessation of the study drug during the study.
17. Participating in another clinical trial with an investigational drug or device concurrently or during the last 30 days.
18. Women of childbearing age who were not practicing reliable contraception and did not have a documented negative pregnancy test or severe noncardiovascular coexisting condition.

19. Severe non-cardiovascular comorbidity with a life expectancy of less than 3 months.

20. With a history of clinically significant drug or alcohol abuse.

21. Inability to understand and/or follow research procedures due to mental, cognitive, or emotional disorders, or to be an unsuitable candidate for the study for any other considered by the investigator.

Definition of acute infection

**Acute respiratory tract infection**	Acute respiratory tract infection was diagnosed by clinical symptoms such as cough and fever, increase in leucocyte count or lymphocyte ratio or neutrophil ratio, typical chest X-ray or CT findings. [1 2 3]
**Acute urinary tract infection**	Acute urinary tract infection was diagnosed by clinical symptoms of urinary frequency, urgency, or dysuria, accompanied by fever or increase in blood leucocyte count, and the presence of leukocyturia or positive nitrate reduction test. [3]
**Acute gastro-enteritis**	Acute gastro-enteritis was diagnosed by clinical symptoms of nausea, vomiting, abdominal pain, or diarrhea, combined with fever or increase in blood or stool leucocyte count. [4 5]

1. Smith CJ, Kishore AK, Vail A, et al. Diagnosis of Stroke-Associated Pneumonia: Recommendations From the Pneumonia in Stroke Consensus Group. *Stroke* 2015;46(8):2335-40. doi: 10.1161/STROKEAHA.115.009617
2. Macfarlane J, Holmes W, Gard P, et al. Prospective study of the incidence, aetiology and outcome of adult lower respiratory tract illness in the community. *Thorax* 2001;56(2):109-14. doi: 10.1136/thorax.56.2.109
3. Yongjian Xu (9th edition 2018). 'Acute upper respiratory tract infection and acute treacheo bronchitis', and 'Pulmonary infectious diseases' in Junbo Ge (eds) *Internal Medicine*. Beijing, People's Medical Press, pp. 14-18 and 41-61
4. Noguera T, Wotring R, Melville CR, et al. Resolution of acute gastroenteritis symptoms in children and adults treated with a novel polyphenol-based prebiotic. *World J Gastroenterol* 2014;20(34):12301-7. doi: 10.3748/wjg.v20.i34.12301
5. Kim YJ, Park KH, Park DA, et al. Guideline for the Antibiotic Use in Acute Gastroenteritis. *Infect Chemother* 2019;51(2):217-43. doi: 10.3947/ic.2019.51.2.217

Definition of stroke events and vascular events

**Stroke**	Acute symptoms and signs of neurologic defect caused by sudden abnormality of the blood supply. Damage of focal or whole brain, spinal or retinal vascular damage, which is related to cerebral circulation disorder.
**Ischaemic stroke**	Definitions: (1) Symptoms or imaging evidence of acute newly onset focal neurologic deficit last for more than 24 hours after excluding other non-ischaemic reasons, such as brain infection, head trauma, brain tumor, epilepsy, severe metabolic diseases, degeneration diseases or adverse effect of medications; or (2) Acute brain or retinal ischaemic event with focal symptoms or signs lasts for less than 24 hours after excluding other causes with imaging evidence of new infarction; or (3) Progression of original vascular ischaemic stroke (NIHSS increased≥4 from baseline score after excluding hemorrhagic transformation or symptomatic intracerebral hemorrhage after cerebral infarction) lasts over 24 hours with new ischaemic lesion on brain MRI or CT. Which would be classified by TOAST etiology standard.
**Transient ischaemic attack**	A brief episode of neurological dysfunction caused by focal brain or retinal ischemia, with clinical symptoms typically lasting less than 24 hours, and without evidence of acute infarction, after excluding other non-ischaemic reasons, such as brain infection, head trauma, brain tumor, epilepsy, severe metabolic diseases, degeneration diseases or adverse effect of medications.
**Hemorrhagic stroke**	Hemorrhagic stroke was defined as focal or whole brain or spine damage caused by non-traumatic bleeding into the brain parenchyma, intraventricular or subarachnoid.
**Myocardial infarction**	Third universal definition of myocardial infarction (Thygesen 2012)<br>The term acute myocardial infarction (MI) should be used when there is evidence of myocardial necrosis in a clinical setting consistent with acute myocardial<br>ischemia. Under these conditions any one of the following criteria meets the diagnosis for MI:<br>1. Detection of a rise and/or fall of cardiac biomarker values [preferably cardiac troponin (cTn)] with at least one value above the 99th percentile upper reference limit (URL) and with at least one of the following:<br>(1) Symptoms of ischemia.<br>(2) New or presumed new significant ST-segment–T wave (ST–T) changes or new left bundle branch block (LBBB).<br>(3) Development of pathological Q waves in the ECG.

	(4) Imaging evidence of new loss of viable myocardium or new regional wall motion abnormality<br>(5) Identification of an intracoronary thrombus by angiography or autopsy.<br>2. Cardiac death with symptoms suggestive of myocardial ischemia and presumed new ischaemic ECG changes or new LBBB, but death occurred before cardiac<br>biomarkers were obtained, or before cardiac biomarker values would be increased.<br>3. Percutaneous coronary intervention (PCI) related MI is arbitrarily defined by elevation of cTn values (>5×99th percentile URL) in patients with normal baseline<br>values (≤99th percentile URL) or a rise of cTn values >20% if the baseline values are elevated and are stable or falling. In addition, either (1) symptoms suggestive of myocardial ischemia or (2) new ischaemic ECG changes or (3) angiographic findings consistent with a procedural complication or (4) imaging demonstration of new loss of viable myocardium or new regional wall motion abnormality are required.<br>4. Stent thrombosis associated with MI when detected by coronary angiography or autopsy in the setting of myocardial ischemia and with a rise and/or fall of cardiac<br>biomarker values with at least one value above the 99th percentile URL.<br>5. Coronary artery bypass grafting (CABG) related MI is arbitrarily defined by elevation of cardiac biomarker values (>10×99th percentile URL) in patients with<br>normal baseline cTn values (≤99th percentile URL). In addition, either<br>(1) new pathological Q waves or new LBBB, or<br>(2) angiographic documented new graft or new native coronary artery occlusion, or<br>(3) imaging evidence of new loss of viable myocardium or new regional wall motion abnormality.
**Vascular death**	Vascular death include death due to stroke, cardiac sudden death, death caused by acute myocardial infarction, death caused by heart failure, death caused by pulmonary embolism, death caused by cardial/cerebral interventions or operations (not caused by myocardial infarction) and death caused by other cardiovascular diseases. (Arrhythmia irrelevant to cardiac sudden death, rupture of aortic aneurysm or peripheral artery disease).

	Unexplained death happened within 30 days after stroke, myocardial infarction or cardiovascular/cerebral vascular operation will be considered as stroke, myocardial infarction and accidental death caused by operation separately.

Definition of symptomatic intracranial artery stenosis and symptomatic extracranial artery stenosis

In CHANCE-3, patients at all participating sites underwent a comprehensive assessment of intra−/extracranial arteries within 7 days after enrollment by magnetic resonance angiography as the preferred method, or CT angiography if magnetic resonance imaging was contraindicated or unavailable, and by carotid ultrasound or digital subtraction angiography if CT angiography was also contraindicated or unavailable. Trained experts centrally reviewed and evaluated the imaging data using a web-based electronic data collection system. Experienced assessors with more than 5 years of experience practicing clinical neuroscience, blinded to the study, determined whether an intracranial or extracranial arterial stenosis was symptomatic based on vascular imaging, brain imaging, and clinical presentations. Presence of symptomatic intracranial artery stenosis was defined as ≥50% stenosis or occlusion in any of the 11 major intracranial arteries in MRA/CTA/DSA/CEMRA that is deemed causal for the index ischaemic event: bilateral intracranial internal carotid arteries (ICA), middle cerebral arteries (MCA, M1 and M2), anterior cerebral arteries (ACA, A1 and A2), posterior cerebral arteries (PCA, P1 and P2), vertebral arteries (VA, V4) and basilar artery (BA). [1] The percentage of stenosis was defined by the Warfarin-Aspirin Symptomatic Intracranial Disease method, which was the percent reduction in vessel diameter at the stenotic throat comparing with a proximal normal vessel diameter. [2] Presence of symptomatic extracranial artery stenosis was defined as ≥50% stenosis or occlusion in any of the 4 major extracranial arteries in doppler/CEMRA/CTA/DSA that is deemed causal for the index ischaemic event: bilateral external carotid arteries and vertebral artery. [1]

Reference

1. Wang Y, Zhao X, Liu L, Soo YO, Pu Y, Pan Y, et al. Prevalence and outcomes of symptomatic intracranial large artery stenoses and occlusions in china: The

chinese intracranial atherosclerosis (cicas) study. *Stroke*. 2014;45:663-669

2. Samuels OB, Joseph GJ, Lynn MJ, Smith HA, Chimowitz MI. A standardized method for measuring intracranial arterial stenosis. *AJNR Am J Neuroradiol*. 2000;21:643-646

Table S1: Patients excluded by exclusion criteria.

Exclusion Criteria	n, (%)
1. Malformation, tumor, abscess or other major non-ischemic brain disease (e.g., multiple sclerosis) on baseline head CT or MRI.	12 (3.9)
2. Isolated or pure sensory symptoms (e.g., numbness), isolated visual changes, or isolated dizziness/vertigo without evidence of acute infarction on baseline head CT or MRI. Isolated or pure sensory symptoms (e.g., numbness), isolated visual changes, or isolated dizziness/vertigo without evidence of acute infarction on baseline head CT or MRI.	17 (5.5)
3. Iatrogenic causes (angioplasty or surgery) of stroke or TIA	0
4. Presumed cardiac source of embolus, such as atrial fibrillation or prosthetic cardiac valve.	48 (15.5)
5. A score of ≥ 2 on the modified Rankin scale immediately before the occurrence of the index event.	9 (2.9)
6. Usage of colchicine within 30 days before randomization or planning to take colchicine therapy for other indications	4 (1.3)
7. Known allergy or sensitivity or intolerance to colchicine	2 (0.7)
8. Inflammatory bowel disease (Crohn's or ulcerative colitis) or chronic diarrhea	10 (3.2)
9. Symptomatic peripheral neuropathy or pre-existing progressive neuromuscular disease or with creatine kinase (CK) level > 3 times the upper limit of normal as measured within the past 30 days and determined to be non-transient through repeat testing	8 (2.6)
10. A history of cirrhosis, chronic active hepatitis or severe hepatic disease	3 (0.97)
11. Impaired hepatic (ALT or AST > twice the upper limit of normal range) or kidney (creatinine exceeding 1.5 times of the upper limit of normal range or eGFR less than 50 ml/min) function at randomization	72 (23.3)
12. Anemia (haemoglobin <10g/dL), thrombocytopenia (platelet count $<100\times10^9$/L) or leucopenia (white blood cell $<3\times10^9$/L) at randomization	34 (11.0)
13. In the acute phase of respiratory tract infection, urinary tract infection, and gastro-enteritis, or currently using or planning to receive oral or intravenous anti-infective therapy for any other infection.	65 (21.0)
14. Currently using or planning to begin long-term (>7 days) systemic anti-inflammatory drugs (NSAIDs except for aspirin, oral or intravenous steroid therapy) during the study	9 (2.9)

15. Planning to use moderate or strong CYP3A4 inhibitors (clarithromycin, erythromycin, telithromycin, other macrolide antibiotics, ketoconazole, itraconazole, voriconazole, ritonavir, atazanavir, indinavir, other HIV protease inhibitors, verapamil, diltiazem, quinidine, digoxin, disulfiram, etc.) or P-gp inhibitors (cyclosporine) at randomization	0
16. Planned surgery or interventional treatment requiring cessation of the study drug during the study	8 (2.6)
17. Participating in another clinical trial with an investigational drug or device concurrently or during the last 30 days	1 (0.3)
18. Women of childbearing age who were not practicing reliable contraception and did not have a documented negative pregnancy test or severe noncardiovascular coexisting condition	0
19. Severe non-cardiovascular comorbidity with a life expectancy of less than 3 months	2 (0.7)
20. With a history of clinically significant drug or alcohol abuse	1 (0.3)
21. Inability to understand and/or follow research procedures due to mental, cognitive, or emotional disorders, or to be an unsuitable candidate for the study for any other considered by the investigator	4 (1.3)

Table S2. Secondary prevention treatments during hospitalization .

Variables	Colchicine (n = 4176)	Placebo (n = 4167)
Medicine use during hospitalization		
Singel Antiplatelet	4103 (98.3%)	4087 (98.1%)
Aspirin	1123 (26.9%)	1080 (25.9%)
Clopidogrel	266 (6.4%)	233 (5.6%)
Ticagrelor	6 (0.1%)	4 (0.1%)
Others	90 (2.2%)	96 (2.3%)
Dual antiplatelet therapy	2809 (67.3%)	2836 (68.1%)
Aspirin + Clopidogrel	2718 (65.1%)	2756 (66.1%)
Aspirin + Ticagrelor	31 (0.7%)	31 (0.7%)
Statin	4112 (98.5%)	4109 (98.6%)
Other lipid-lowering agents	309 (7.4%)	343 (8.2%)
Hypoglycemic agents *	1086 (81.6%)	1113 (82.7%)
Antihypertensive agents †	2219 (69.9%)	2228 (69.1%)

Data are n (%).

* The number of patients with diabetes in the colchicine and placebo group were 1331 and 1346, respectively.

† The number of patients with hypertension in the colchicine and placebo group were 3175 and 3223, respectively.

Table S3: Prohibited concomitant medications within 90 days.

Category	Colchicine (n = 4176)	Placebo (n = 4167)	Temporary discontinuation	Permanent discontinuation	No discontinuation		
					No.	SAE	Timing and duration of prohibited medication
Strong CYP3A4 inhibitors or P-glycoprotein inhibitors	3 (0.1%)	5 (0.1%)	0	1 (12.5%)	7 (87.5%)	0	• All 7 patients had one dose of prohibited medication during hospitalization.
Moderate CYP3A4 inhibitors	8 (0.2%)	12 (0.3%)	0	1 (5.0%)	19 (95.0%)	0	• All 19 patients had prohibited medication for a medium and IQR of 3 (1-5) days during hospitalization:<br>— 7 patients had it for once;<br>— 12 patients had it for a medium and IQR of 5 (3-6) days.
Other anti-inflammatory medications (except for aspirin)	96 (2.3%)	112 (2.7%)	10 (4.8%)	17 (8.2%)	181 (87.0%)	2 (1.1%)	• 175 (96.7%) patients had prohibited medications for a medium and IQR of 1 (1-4) days during hospitalization:<br>— 98 patients had it for once；<br>— 77 patients had it for a medium and IQR of 4 (2-7) days.<br>• 6 (3.3%) patients had prohibited medications after discharge:<br>— 3 patients had it for once;<br>— 3 patients had it for 2, 7 and 8 days respectively.

Data are n (%). SAE= serious adverse events.

Table S4: Hemorrhagic stroke and Modified Rankin scale score within 90 days.

Outcomes	No. of events (%) Colchicine (n=4176)	Placebo (n=4167)	Odds Ratio (95% CI)	P Value
Hemorrhagic stroke	8 (0.2%)	7 (0.2%)	1.14 (0.41 to 3.15)	0.80
mRS 3-6	186 (4.5%)	199 (4.8%)	0.93 (0.76 to 1.14)	0.49
Ordinary mRS score				
0 (no symptoms at all)	2,290 (54.8%)	2,265 (54.4%)	0.98 (0.90 to 1.07)	0.67
1 (no significant disability despite symptoms)	1,451 (34.7%)	1,462 (35.1%)		
2 (slight disability)	249 (6.0%)	241 (5.8%)		
3 (moderate disability requiring some help)	93 (2.2%)	85 (2.0%)		
4 (moderate-severe disability requiring assistance with daily living)	48 (1.1%)	56 (1.3%)		
5 (severe disability, bed bound, and incontinent)	11 (0.3%)	9 (0.2%)		
6 (dead)	34 (0.8%)	49 (1.2%)		

Table S5: Efficacy outcomes in per-protocol population.

Efficacy Outcomes	Colchicine (N=3838)		Placebo (N=3795)		Hazard Ratio or Odds Ratio (95% CI) *	P value
	No. (%)	Event Risk†	No. (%)	Event Risk†		
Primary outcome						
Stroke	235 (6.1%)	6.1	229 (6.0%)	6.0	1.02 (0.85 to 1.22)	0.84
Secondary outcomes						
Vascular events‡	263 (6.9%)	6.9	257 (6.8%)	6.8	1.01 (0.85 to 1.20)	0.87
Ischaemic stroke	230 (6.0%)	6.0	226 (6.0%)	6.0	1.01 (0.84 to 1.21)	0.91
Stroke or TIA	252 (6.6%)	6.6	246 (6.5%)	6.5	1.02 (0.85 to 1.21)	0.86
Poor functional outcome§	373 (9.7%)		370 (9.7%)		1.00 (0.86 to 1.17)	0.98
Ordinal stroke or TIA¶					0.99 (0.83 to 1.18)	0.91
Fatal stroke: score of 6 on mRS	6 (0.2%)		11 (0.3%)			
Severe stroke: score of 4 or 5 on mRS	38 (1.0%)		42 (1.1%)			
Moderate stroke: score of 2 or 3 on mRS	90 (2.3%)		86 (2.3%)			
Mild stroke: score of 0 or 1 on mRS	101 (2.6%)		90 (2.4%)			
TIA	17 (0.4%)		17 (0.4%)			
No stroke or TIA	3586 (93.4%)		3549 (93.5%)			

Data are n (%). TIA= Transient Ischaemic Attack. mRS=modified Rankin Scale.

* The odds ratio is shown for poor functional outcome; common odds ratio is shown for ordinal stroke or TIA; hazard ratios are shown for other outcomes.

† Event risk are estimated by Kaplan–Meier method.

‡ Vascular events were a composite of ischaemic stroke, hemorrhagic stroke, TIA, myocardial infarction, or death from vascular causes.

§ Defined by the modified Rankin scale (mRS) score greater than 1. The mRS Scores range from 0 to 6, with a score of 0 indicating no symptoms; a score of 5 indicating severe disability; and a score of 6 indicating death.

¶Severity was measured with the use of a six-level ordinal scale that incorporates subsequent stroke or TIA events and the score on the modified Rankin scale at 3 months

Table S6: Adverse events and serious adverse events within 90 days in patients with minor-to-moderate ischaemic stroke or TIA and a high-sensitivity C-reactive protein ≥2 mg/L assigned to receive colchicine or placebo.

**Safety outcomes**	**Colchicine (n = 4176)**	**Placebo (n = 4167)**	**Hazard Ratio**
Primary safety outcome			
Any serious adverse event	91 (2.2%)	88 (2.1%)	1.03 (0.77 to 1.38)
Other safety outcomes			
Serious adverse event			
Death	34 (0.8%)	45 (1.1%)	0.75 (0.48 to 1.17)
Cardiovascular death	19 (0.5%)	27 (0.7%)	0.96 (0.53 to 1.73)
Non-cardiovascular death	15 (0.4%)	18 (0.4%)	0.83 (0.42 to 1.65)
Gastrointestinal event	8 (0.2%)	7 (0.2%)	1.14 (0.41 to 3.14)
Infection	4 (0.1%)	5 (0.1%)	0.80 (0.21 to 2.97)
Pneumonia	16 (0.4%)	7 (0.2%)	2.28 (0.94 to 5.54)
Adverse events	910 (21.8%)	888 (21.3%)	1.03 (0.93 to 1.12)
Gastrointestinal event	173 (4.1%)	150 (3.6%)	1.16 (0.93 to 1.44)
Diarrhea	71 (1.7%)	30 (0.7%)	2.37 (1.55 to 3.63)
Flatulence	21 (0.5%)	10 (0.2%)	2.10 (0.99 to 4.45)
Constipation	30 (0.7%)	45 (1.1%)	0.66 (0.42 to 1.05)
Dyspepsia	23 (0.6%)	29 (0.7%)	0.79 (0.46 to 1.37)
Gastrointestinal hemorrhage	9 (0.2%)	15 (0.4%)	0.60 (0.26 to 1.37)
Others	25 (0.6%)	26 (0.6%)	0.96 (0.55 to 1.66)
Anemia	27 (0.6%)	20 (0.5%)	1.35 (0.76 to 2.40)
Leukopenia	5 (0.1%)	0 (0.0%)	
Thrombocytopenia	8 (0.2%)	3 (0.1%)	2.65 (0.70 to 9.99)
Myopathy	0	0	
Increased CK levels†	0	0	
Increased ALT or AST levels	29 (0.7%)	19 (0.5%)	1.52 (0.85 to 2.72)
Abnormal hepatic function‡	12 (0.3%)	3 (0.1%)	3.99 (1.13 to 14.14)

Data are n (%). CK= Creatine Kinase. ALT= Alanine Transaminase. AST= Aspertate Aminotransferase

* P value was calculated by Fisher's exact test. †⩾ 5 times the upper limit of normal. ‡ ALT or AST ⩾ 3 times the upper limit of the normal range.

Table S7: Number of patients with serious adverse events* (by system organ class) up to 3-month visit.

System organ class	Colchicine (n = 4176)	Placebo (n = 4167)
Cardiac disorders	21 (0.5%)	13 (0.3%)
Endocrine disorders	2 (0.0%)	1 (0.0%)
Gastrointestinal disorders	8 (0.2%)	7 (0.2%)
Diarrhea	1 (0.02%)	1 (0.02%)
Constipation	1 (0.02%)	0 (0%)
Intestinal obstruction	2 (0.5%)	1 (0.02%)
Vomiting	0 (0%)	1 (0.02%)
Gastrointestinal hemorrhage	1 (0.02%)	2 (0.05%)
General disorders and administration site conditions	8 (0.2%)	12 (0.3%)
Hepatobiliary disorders	1 (0.0%)	2 (0.0%)
Infections and infestations	4 (0.1%)	6 (0.1%)
Injury, poisoning and procedural complications	1 (0.0%)	2 (0.0%)
Metabolism and nutrition disorders	0 (0.0%)	1 (0.0%)
Musculoskeletal and connective tissue disorders	0 (0.0%)	2 (0.0%)
Neoplasms benign, malignant and unspecified	7 (0.2%)	4 (0.1%)
Nervous system disorders	33 (0.8%)	33 (0.8%)
Renal and urinary disorders	2 (0.0%)	2 (0.0%)
Respiratory, thoracic and mediastinal disorders	19 (0.5%)	10 (0.2%)
Surgical and medical procedures	1 (0.0%)	3 (0.1%)
Vascular disorders	1 (0.0%)	0 (0.0%)
Death	34 (0.8%)	45 (1.1%)
Cardiovascular death	19 (0.5%)	27 (0.7%)

Data are n (%).

* Patients with multiple events of one type were counted once. Includes serious adverse events with an onset date on or after the date of the first dose and up to the date of the last dose of study medication.

Table S8: Number of patients with adverse events (by system organ class) up to 3-month visit.*

System organ class	Colchicine (n = 4176)	Placebo (n = 4167)
Blood and lymphatic system disorders	38 (0.9%)	23 (0.6%)
Cardiac disorders	75 (1.8%)	81 (1.9%)
Ear and labyrinth disorders	2 (0.0%)	1 (0.0%)
Endocrine disorders	2 (0.0%)	6 (0.1%)
Eye disorders	3 (0.1%)	11 (0.3%)
Gastrointestinal disorders	173 (4.1%)	150 (3.6%)
General disorders and administration site conditions	63 (1.5%)	84 (2.0%)
Hepatobiliary disorders	57 (1.4%)	48 (1.2%)
Immune system disorders	11 (0.3%)	10 (0.2%)
Infections and infestations	52 (1.2%)	39 (0.9%)
Injury, poisoning and procedural complications	2 (0.0%)	7 (0.2%)
Investigations	35 (0.8%)	29 (0.7%)
Metabolism and nutrition disorders	151 (3.6%)	163 (3.9%)
Musculoskeletal and connective tissue disorders	29 (0.7%)	34 (0.8%)
Neoplasms benign, malignant and unspecified	5 (0.1%)	1 (0.0%)
Nervous system disorders	223 (5.3%)	200 (4.8%)
Psychiatric disorders	52 (1.2%)	43 (1.0%)
Renal and urinary disorders	60 (1.4%)	80 (1.9%)
Reproductive system and breast disorders	8 (0.2%)	4 (0.1%)
Respiratory, thoracic and mediastinal disorders	129 (3.1%)	124 (3.0%)
Skin and subcutaneous tissue disorders	17 (0.4%)	17 (0.4%)
Surgical and medical procedures	0 (0.0%)	1 (0.0%)
Vascular disorders	22 (0.5%)	23 (0.6%)

Data are n (%).

* Patients with multiple events of one type were counted once. Includes adverse events with an onset date on or after the date of the first dose and up to the date of the last dose of study medication.

Table S9: Number of patients with adverse events or serious adverse events leading to premature discontinuation of study agents by system organ class up to 3-month visit.

**System organ class**	**Colchicine (n = 4176)**	**Placebo (n = 4167)**
**Total**	**68 (1.6%)**	**62 (1.5%)**
Blood and lymphatic system disorders	3 (0.1%)	0 (0.0%)
Cardiac disorders	6 (0.1%)	2 (0.0%)
Endocrine disorders	1 (0.0%)	0 (0.0%)
Gastrointestinal disorders	31 (0.7%)	23 (0.6%)
General disorders and administration site conditions	1 (0.0%)	2 (0.0%)
Hepatobiliary disorders	2 (0.0%)	0 (0.0%)
Immune system disorders	1 (0.0%)	1 (0.0%)
Infections and infestations	1 (0.0%)	2 (0.0%)
Injury, poisoning and procedural complications	0 (0.0%)	1 (0.0%)
Investigations	1 (0.0%)	0 (0.0%)
Metabolism and nutrition disorders	1 (0.0%)	1 (0.0%)
Musculoskeletal and connective tissue disorders	0 (0.0%)	1 (0.0%)
Neoplasms benign, malignant and unspecified	2 (0.0%)	1 (0.0%)
Nervous system disorders	12 (0.3%)	21 (0.5%)
Renal and urinary disorders	0 (0.0%)	2 (0.0%)
Reproductive system and breast disorders	0 (0.0%)	1 (0.0%)
Respiratory, thoracic and mediastinal disorders	3 (0.1%)	4 (0.1%)
Skin and subcutaneous tissue disorders	3 (0.1%)	0 (0.0%)

Data are n (%).

Table S10: Adherence to study agents and secondary prevention treatments used within 90 days among patients had serious adverse events.

Agents or treatments	Colchicine (n = 91)	Placebo (n = 88)
Adherence to study agents *	61 (67.0%)	50 (56.8%)
Secondary prevention treatments used during hospitalization		
Antiplatelet	85 (93.4%)	80 (90.9%)
Dual antiplatelet therapy	59 (64.8%)	48 (54.5%)
Statin	88 (96.7%)	83 (94.3%)
Other Lipid-lowering agents	6 (6.6%)	8 (9.1%)
Hypoglycemic agents †	32 (80.0%)	38 (84.4%)
Antihypertensive agents ‡	53 (67.9%)	50 (66.7%)
Secondary prevention treatments used at 90-day follow-up		
Antiplatelet	59 (64.8%)	44 (50.0%)
Dual antiplatelet therapy	26 (28.6%)	25 (28.4%)
Statin	61 (67.0%)	48 (54.5%)
Other lipid-lowering agents	5 (5.5%)	4 (4.5%)
Hypoglycemic agents †	21 (52.5%)	14 (31.1%)
Antihypertensive agents ‡	41 (52.6%)	33 (44.0%)

Data are n (%).

* The definition for treatment adherence: Pill counts of the pills taken during study period in the range of 80-120%.

† The number of patients with diabetes in the colchicine and placebo group were 40 and 45, respectively.

‡ The number of patients with hypertension in the colchicine and placebo group were 78 and 75, respectively.

Table S11: Adherence to study agents and secondary prevention treatments used within 90 days among patients had adverse events.

Agents or treatments	Colchicine (n = 910)	Placebo (n = 888)
Adherence to study agents *	821 (90.2%)	803 (90.4%)
Secondary prevention treatments used during hospitalization		
Antiplatelet	884 (97.1%)	869 (97.9%)
Dual antiplatelet therapy	566 (62.2%)	551 (62.0%)
Statin	887 (97.5%)	878 (98.9%)
Other lipid-lowering agents	88 (9.7%)	94 (10.6%)
Hypoglycemic agents †	231 (83.1%)	254 (80.1%)
Antihypertensive agents ‡	509 (72.4%)	507 (72.4%)
Secondary prevention treatments used at 90-day follow-up		
Antiplatelet	851 (93.5%)	825 (92.9%)
Dual antiplatelet therapy	383 (42.1%)	351 (39.5%)
Statin	856 (94.1%)	827 (93.1%)
Other lipid-lowering agents	48 (5.3%)	53 (6.0%)
Hypoglycemic agents †	204 (73.4%)	237 (74.8%)
Antihypertensive agents ‡	525 (74.7%)	511 (73.0%)

Data are n (%).

* The definition for treatment adherence: Pill counts of the pills taken during study period in the range of 80-120%.

† The number of patients with diabetes in the colchicine and placebo group were 278 and 317, respectively.

‡ The number of patients with hypertension in the colchicine and placebo group were 703 and 700, respectively.

Table S12: Secondary prevention treatments used within 90 days among patients had premature discontinuation of study agents.

Agents or treatments	Colchicine (n = 198)	Placebo (n = 206)
Secondary prevention treatments used during hospitalization		
Antiplatelet	187 (94.4%)	196 (95.1%)
Dual antiplatelet therapy	146 (73.7%)	145 (70.4%)
Statin	194 (98.0%)	200 (97.1%)
Other Lipid-lowering agents	20 (10.1%)	21 (10.2%)
Hypoglycemic agents *	58 (76.3%)	57 (76.0%)
Antihypertensive agents †	95 (62.5%)	101 (65.2%)
Secondary prevention treatments used at 90-day follow-up		
Antiplatelet	161 (81.3%)	160 (77.7%)
Dual antiplatelet therapy	95 (48.0%)	88 (42.7%)
Statin	162 (81.8%)	164 (79.6%)
Other lipid-lowering agents	14 (7.1%)	11 (5.3%)
Hypoglycemic agents *	51 (67.1%)	41 (54.7%)
Antihypertensive agents†	89 (58.6%)	93 (60.0%)

Data are n (%).

* The number of patients with diabetes in the colchicine and placebo group were 76 and 75, respectively.

† The number of patients with hypertension in the colchicine and placebo group were 152 and 155, respectively.

Figure S1: Graph of model estimating hazard ratio for any new stroke within 90 days in colchicine group compared to placebo group by age as a continuous variable with 95% confidence intervals. The blue line indicates the point estimate of the effect of colchicine compared to placebo (reference group) for the specified age. The blue band indicates the 95% confidence interval of the predicted treatment effect. Hazard Ratio >1 indicates higher risk that colchicine-treated patients have a new stroke within 90 days compared to the placebo-treated patients when at the specified age. Range of age was 40 to 95.

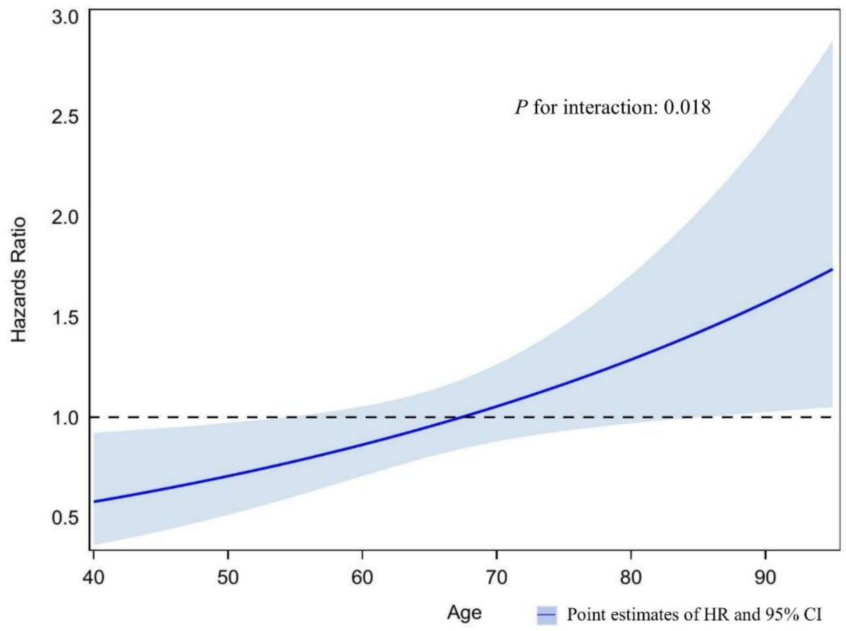

Figure S2: Hazard ratios and 95% confidence intervals by trial center.

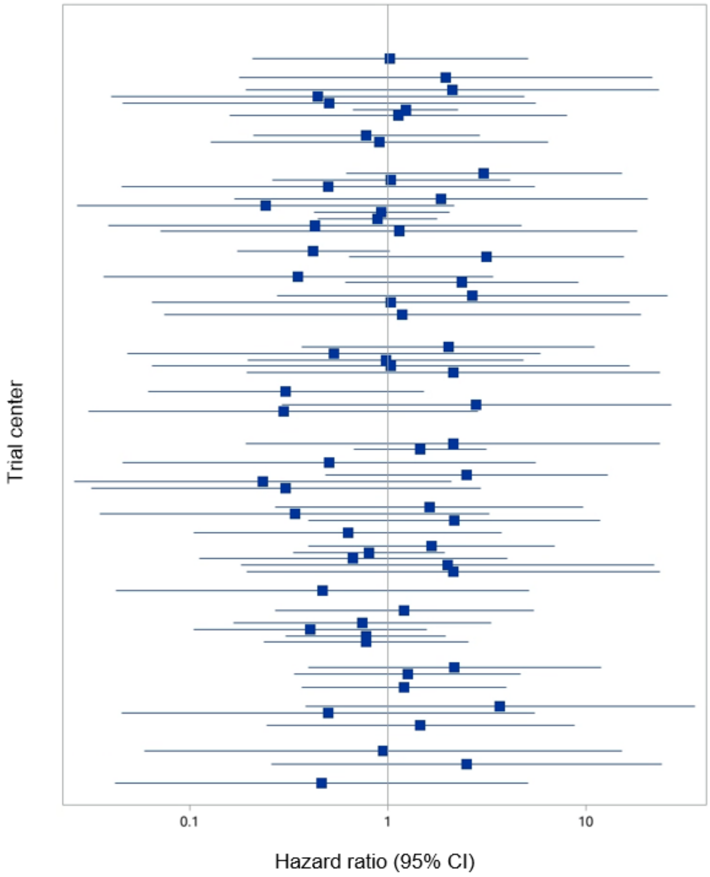

Supplement: Supplementary file 1 — Web appendix: Extra material supplied by authors [file liji079061.ww.pdf]
